# Effects of Exercise on Depression, Anxiety, Cognitive Control, Craving, Physical Fitness and Quality of Life in Methamphetamine-Dependent Patients

**DOI:** 10.3389/fpsyt.2019.00999

**Published:** 2020-01-28

**Authors:** Junhao Huang, Yuqing Zheng, Dongdong Gao, Min Hu, Tifei Yuan

**Affiliations:** ^1^ Guangdong Provincial Key Laboratory of Sports and Health Promotion, Scientific Research Center, Guangzhou Sport University, Guangzhou, China; ^2^ Shanghai Key Laboratory of Psychotic Disorders, Shanghai Mental Health Center, Shanghai Jiao Tong University School of Medicine, Shanghai, China; ^3^ Co-Innovation Center of Neuroregeneration, Nantong University, Nantong, China

**Keywords:** methamphetamine addiction, exercise, depression, anxiety, cognition, craving, physical fitness, quality of life

## Abstract

Methamphetamine (MA) abuse results in a variety of harmful changes in mood states and cognitive function, together with declined physical health and quality of life. Recent studies highlighted the therapeutic potential of physical exercise on MA addiction. Physical exercise improves emotional state and general health conditions, enhances cognitive function, reduces relapse rate, and facilitates abstinence, thereby improves the overall quality of life of the drug users. This review summarizes the present situation of physical exercise on MA-dependent patients with both animal and clinical population results.

## Introduction

Drug use is extremely harmful, with about 3.1 million drug users suffering from drug abuse, which means they may need treatment. Of these, only 1/6 of drug users received treatment, a relatively low proportion that has remained unchanged in recent years. According to the World Health Organization, about 450,000 people died from drug abuse in 2015. Of these deaths, 167,750 were directly related to drug abuse, mainly due to excessive drug use (1). Other deaths were indirectly associated with drug use, including deaths from HIV and hepatitis C as a result of unsafe injection practices. Moreover, many countries are still unable to provide adequate treatment and health-care services to reduce the negative health consequences of drug abuse.

During the period from 2013 to 2016, the supply of methamphetamine (MA) increased in North America, which became the second largest drug threat in the United States in 2016, after heroin (1). Over these years, amphetamine has dominated the synthetic drug markets in the Near East and Middle East and Western and Central Europe, and recently in North Africa and North America. MA abuse impairs physical and mental health of individuals and may lead to depression, mental illness, and behavioral disorders, which may eventually lead to substance dependence, overdose, and suicide (2, 3). The prevalence of MA is extremely high, especially among young people, so the use of MA is a serious global public health problem, and abuse of MA has a serious impact on a country’s personal, social, economic, environmental, and health systems.

Currently, drug therapy, cognitive behavioral therapy (CBT), and psychotherapy have achieved certain therapeutic effects in management of MA use disorder. However, these therapies also have some prominent negative effects, including high cost of drug development and some adverse drug reactions such as drug dependence. More importantly, there are no medications that have been identified as clearly effective for treatment of the disorder. The US Food and Drug Administration has approved no medication for the treatment of MA use disorder. CBT and psychotherapy also have some shortcomings, such as poor compliance and lack of effective evaluation. Therefore, there is a need to further seek new ways to treat MA addiction. Recent evidence showed that the damage caused by drug abuse can be reversed by physical exercise (4). The present review summarized the current evidence of exercise on reducing depression and anxiety status, improving cognitive function, reducing craving, enhancing physical fitness, and quality of life in MA-dependent patients.

## Effects of Exercise on Mood States of Methamphetamine-Dependent Patients

MA binds to dopamine, norepinephrine, and serotonin transporters in neurons and results in rapid accumulation of monoamine neurotransmitters in synapses of the brain (5). MA abuse affects the brain reward pathway by releasing neurotransmitters to induce euphoria (6). Long-term MA abuse severely damages the structure and function of the monoamine transmitter system in MA-dependent patients, leading to the depletion of monoamine neurotransmitters (7), and disorders of release of neurotransmitters such as dopamine (DA), norepinephrine (NE), and 5-hydroxytryptamine (5-HT) (8, 9). Concretely, chronic abuse of MA can deplete dopamine reserves in the brain and reduce dopamine receptor availability (10). The changes of DA levels in the brain regulate people’s depression, anxiety and other mood states, which will produce reward effect, drug craving and so on. Moreover, 5-HT and NE neurotransmitters are also closely related to MA dependence and play an important role in regulating synaptic plasticity and improving mood states (11, 12). MA also impairs motor and executive functions, as well as episodic memory defects, which are usually associated with anxiety and depression (13). London ED et al. used fluorodeoxyglucose positron emission tomography to measure mood and cerebral glucose metabolism during performance of a vigilance task, they found that MA users have abnormalities in brain regions implicated in mood disorders (14). In clinical population, the severity of MA use disorder is related to the increased incidence of depression and anxiety (15).

MA abstinence syndromes include a desire for drugs, lack of pleasure, irritability, lack of concentration, lethargy, hypothermia, and even suicidal psychiatric characteristics (16–18). In addition, emotional disorders such as depression and anxiety are closely related to the relapse and rehabilitation of MA-dependent people, especially in the early stages of rehabilitation withdrawal, which significantly contributes to relapse (19, 20). One study showed that physical exercise may reverse the physiological and nerve injury caused by MA exposure, and increase the release of brain-derived neurotrophic factor (BDNF), which underlies the antidepressant-like effect (21). Aerobic exercise intervention (8–12 weeks) could provide positive help to groups suffering from emotional disorders (22, 23), and this benefit was also found in drug-dependent groups (24–28).

Haglund et al. found that after 8 weeks of moderate-intensity structured exercise (aerobic + resistance training), participants who received exercise intervention had significantly lower depression scores than those who were randomly assigned to the health education group, and those who took the most frequent exercise had the best results. Further studies had concluded that the effect of exercise is better in the early stage of addiction withdrawal, exercise can effectively treat the depression symptoms of dependent individuals in the early stage of addiction. In addition, exercise therapy seems to be particularly beneficial to individuals with severe medical, mental, and addictive diseases (27). Similarly, Rawson *et al*. found that the 8-week exercise plan significantly improved the depression and anxiety symptoms of newly abstinent MA-dependent patients in hospital, and during the 8-week follow-up, the depression and anxiety levels in the exercise group were significantly lower than those in the health education control group (28).

In addition, the therapeutic effect of physical and mental exercise programs such as Tai Chi, Qigong, and Yoga have similar therapeutic effects as to aerobic exercise, and also has a good effect on depression and anxiety of MA-dependent patients (29, 30). Zhu et al. found that relaxation training related to Tai Chi reduced anxiety by helping individuals to increase their self-awareness and internal attention (30). The similar effect has been found in animal experiments, and physical exercise may improve the consequences of some withdrawal behaviors of MA use disorder. For example, regular swimming could reduce voluntary MA consumption in animal models by reducing anxiety and depression in MA withdrawal rats (31).

## Effect of Exercise on Cognition of Methamphetamine-Dependent Patients

Chronic drug abuse accompanies reduced ability of inhibitory control. Kalivas and Volkow (32), as well as Dawe et al. (33), believe that inhibitory control deficits in cognitive control are important markers of drug dependence. Among different types of drug users, the inhibition control of stimulant users (such as cocaine, MA, etc.) showed the characteristics of higher response error rate, longer reaction time, and lower inhibitory ability, while compared to opioid type substance users (34, 35). Al-Zahrani and Elsayed (36) also found that MA users had longer response to Stroop tasks than alcohol and opioid users. In addition, MA-dependent patients responded to interference stimulation lower than the control group, but were not affected by non-interference stimulation. ED London *et al*. found that patients who abuse MA often display several signs of cortical striatum dysfunction, including abnormal gray- and white-matter integrity, monoamine neurotransmitter system deficiencies, neuroinflammation, poor neuronal integrity, and aberrant patterns of brain connectivity and function, both when engaged in cognitive tasks and at rest. More importantly, these neural abnormalities were found to be linked with certain addiction-related phenotypes that may influence treatment response (e.g., poor self-control, cognitive inflexibility, maladaptive decision-making) (37).

In one meta-analysis, Scott et al. found that MA-dependent persons have performed poorly on tasks that require inhibitory control and refresh capabilities, such as the Stroop task and the Wisconsin Test (38). The researchers also found that 27 drug users who had quit drug addiction for more than a year and 38 new drug users and non-user healthy controls, respectively, completed the Stroop attention test. The results showed that there was a significant positive correlation between Stroop reaction time interference and drug use time and withdrawal time, which indicated that the longer withdrawal time, the more recovery of inhibitory ability (39). Many studies have shown that MA-dependent patients show higher error rate and slower response time in Stroop tasks than healthy people (40, 41). Monterosso JR *et al*. used the stop-signal task to measure response inhibition in 11 MA users and two groups of control subjects who did not use MA, found the stop-signal reaction time (SSRT) was significantly longer for MA users than for either control group (42). However, some studies failed to identify differences in the inhibitory ability of drug dependent individuals compared with healthy controls. Vander Plas et al. compared the stop-signal reaction of alcohol-, cocaine-, and MA-dependent people as a criterion of inhibitory ability, and found no inhibitory functional deficits (43). Moreover, the results of Stroop tasks are not consistent (40, 44).

Physical exercise improves the cognition function in both normal and cognitively impaired adults (45, 46). Although the MA users after treatment still have considerable cognitive impairment, physical exercise can improve the recovery of the damage (4, 47) by the effect of a potential neurobiological process, such as a dopamine activity. The effect of exercise on the inhibitory ability of MA-dependent patients has been reported, and the effects of various exercise intensities on the inhibitory ability are also different. Some recent experimental results show that moderate-intensity aerobic exercise has the most beneficial effect on the improvement of inhibitory ability (48–50). The study of event-related potential (ERP) also found that the accuracy of inhibition control and behavioral performance were improved after moderate-intensity aerobic exercise, such as shorter reaction time (46, 51, 52). Wang et al. found a dose-response relationship between different exercise intensities and inhibition control in MA-dependent patients. The ERPs were recorded by Go/No Go task, and the inverted U-shaped relationship between exercise intensity and inhibition control was observed. The moderate-intensity exercise group showed shorter Go reaction time, increased No Go accuracy, and larger No Go-N2 amplitude (52). Whereas, some studies have shown that low-intensity aerobic exercise had no effect on the inhibition ability, while high-intensity exercise reduced the performance of inhibition tasks (53).

In addition, there is a difference in the effects of acute and chronic exercise on the inhibition ability. The study shows that an acute aerobic exercise can increase the inhibitory capacity. In the context of non-drug intervention, behavior and electrophysiological measurements were carried out by using Go/No Go tasks of standard cues and meth-related clues to examine the effects of acute exercise on related craving and inhibition control of MA users. Acute exercise improved the inhibition control of MA users and increased neurophysiological activity related to the behavioral task (54). Chen et al. found that after 20 min of acute aerobic exercise, the performance of inhibitory tasks was significantly improved in preadolescent children (55). A meta-analysis also found that acute aerobic exercise has a beneficial effect on inhibitory ability (56). In addition, the favorable effect of chronic aerobic exercise on inhibitory ability has been reported. For example, a 12-week moderate-intensity aerobic exercise training may have a beneficial effect on blood lipid peroxide and cognitive function in MA-dependent patients (57).

## Effect of Exercise on Craving of Methamphetamine-Dependent Patients

Compared with heroin users, MA users have more severe craving and anxiety symptoms for drugs (58). In order to maintain a certain sense of pleasure, it is necessary to further increase the use of drugs to make up for the reduction of dopamine release. Some studies have also shown that the homeostatic imbalance of dopamine in midbrain caused by drug addiction is related to the decrease of dopamine D2 receptor level in striatum. Lee B *et al*. measured the availability of dopamine D2/D3 receptor in striatum by Barratt Impulsiveness Scale (version 11, BIS-11) and positron emission tomography with [18F] fallacy, found that low striatal D2/D3 receptor availability may mediate impulsive temperament and thereby influence addiction (59). Animal study have shown that 6 weeks of treadmill exercise can up-regulate the level of dopamine D2 receptor in striatum and regulate the homeostasis of dopamine in midbrain. (60). Exercise enhances the ability of dopamine signal transduction, especially in the reward pathway, to reduce overuse of drugs. O’dell et al. (61) also have shown that running wheel exercise can ameliorate MA-induced damage to dopamine and serotonin terminals and regulate the normalization of reward pathways (61). In addition, a study has shown that chronic wheel running-induced reduction of extinction and reinstatement of MA seeking in MA-dependent rats is associated with reduced numbers of periaqueductal gray dopamine neurons (62).

Therefore, exercise may improve the function of dopamine system by promoting the increase of dopamine release and increasing the expression of dopamine D2 receptor, so as to regulate the normalization of reward system, alleviate withdrawal symptoms, improve the success rate of withdrawal, and reduce or delay the occurrence of relapse.

The benefits of aerobic exercise on craving of MA-dependent patients have been observed in human studies. One study showed that the self-reported MA craving significantly decreased during exercise, immediately after exercise and 50 min after exercise, and the craving scores at these time points after exercise were lower than those in control group. In the study of abstinence MA-dependent patients, it was only found that 20 min of moderate-intensity acute aerobic exercise could temporarily relief the craving of MA-dependent patients (54). In the follow-up study, the dose-response relationship between various exercise intensities (low, moderate, and high intensity) and MA craving were compared. It was also found that the decrease of self-reported MA craving score in moderate- and high-intensity exercise groups was greater than that in low-intensity and control group during acute exercise, immediately after exercise, and 50 min after exercise (52). The similar effect was observed in long-term moderate-intensity aerobic exercise. The results showed that compared with the control group, the MA craving level of the exercise group decreased after 6 weeks of exercise, and maintained a downward trend until the end of treatment (63). In addition, Rawson found that the use of MA was also significantly decreased after 8 weeks of aerobic exercise intervention (64). However, Clinical study from Trivedi et al. have showed that dosed exercise intervention was not superior to health education intervention in reducing stimulant use days, but exercise may improve outcomes for stimulant users with better adherence to an exercise dose (65). Therefore, an acute moderate-intensity exercise and a long-term exercise have a good effect on reducing the craving of MA-dependent patients, and long-term exercise has a sustained therapeutic effect.

## Effects of Exercise on Physical Health and Quality of Life of Methamphetamine-Dependent Patients

The typical sleepy lifestyle caused by MA use disorder, and the related neurosuppression and the direct effects of drugs on the brain and body, such as body mass index (BMI) loss, muscle atrophy, and posture balance, are very important issues (13). The physical health status of MA-dependent patients is significantly decreased, while long-term aerobic exercise could provide some healthy benefits to MA-dependent patients. Studies have shown that 8-week aerobic exercise could lead to of the improvements in physical fitness in MA-dependent patients, including aerobic exercise ability, muscle strength and endurance, changes in body composition, and heart rate variability (66, 67). These changes in physical fitness provide a basis for MA-dependent populations to restore health and avoid relapse. Aerobic exercise intervention improves the aerobic capacity of MA-dependent individuals, which reduces the risk of chronic diseases such as heart disease, stroke, diabetes, hypertension, and cerebrovascular dysfunction in MA-dependent individuals due to long-term exposure to MA (3, 68, 69)

In addition, Zhu et al. (30) used body fat, BMI, blood pressure, heart rate, vital capacity, hand grip strength, sit-and-reach test, and one leg standing with eyes closed as measurement indexes of physical fitness for MA-dependent patients. Compared with the standard treatment group, the balance, hand grip strength, one leg standing with eyes closed, and vital capacity of the Tai Chi exercise group were significantly improved. It is also shown that exercise can normalize BMI in MA-dependent people, although this trend is not statistically significant. In clinical manifestations, the normalization of BMI is important for MA-dependent patients, because chronic MA use can inhibit appetite, resulting in abnormal weight loss and below the normal BMI value. This may lead to second complications, such as malnutrition, hunger, decreased bone mineral density, weakened immune system, anemia, hair loss, dry skin, and sterility (68). These isolated complications may have a negative impact on the quality of life of MA-dependent patients, and are prone to other MA-related complications. For example, individuals with low bone quality may have balance disorders, and when they fall, the bone is more likely to break. Given the known benefits of exercise for muscle mass, strength, and function (70), it seems to be a feasible strategy for the treatment of complications associated with BMI reduction.

Studies have shown that MA abuse is associated with withdrawal from social interaction, so there is a need to develop strategies to promote community integration (71). It is reported that exercise can improve self-esteem and cognitive function, reduce social withdrawal, and improve the quality of life (72). Zhu et al. used drug addiction/dependence (QOL-DA) to assess the quality of life of MA-dependent individuals. The scale includes issues related to physiology, psychology, symptoms, and society. The results showed that it was very effective to use exercise to improve the quality of life and social interaction among MA-dependent patients (30) (**Table S1**).

## Conclusion

The purpose of this review is to evaluate the effectiveness of exercise, including reducing the emotional states of depression and anxiety in MA-dependent patients, improving cognitive control ability, reducing the craving for drugs, and improving their physical health and quality of life. The results showed that exercise intervention is effective to the rehabilitation process of MA-dependent patients.

To sum up, the long-term aerobic exercise has a good effect on MA-dependent patients. It can be used as an effective auxiliary treatment for drug users to abstain from drug addiction. Combined with drug therapy, CBT, and psychotherapy, it can better promote the withdrawal and physical recovery of MA-dependent patients, and has a lasting effect on preventing drug addiction. According to the psychological characteristics and physical health status of different drug users, the future research should combine different exercise patterns with each other, so as to better promote the drug addiction withdrawal and rehabilitation treatment of drug-dependent patients.

## Author Contributions

All authors designed the study together. JH and YZ wrote the manuscript. TY, MH and DG participated in the revision and guidance.

## Funding

The present study was supported by the National Natural Science Foundation of China (grant numbers 31771215, 31600969, 81822017). TY is also sponsored by the Science and Technology Commission of Shanghai Municipality (18QA1403700, 18JC1420304, 18140901700), Shanghai Municipal Education Commission - Gaofeng Clinical Medicine Grant Support (20181715), Hundred-talent Fund from Shanghai Municipal Commission of Health (2018BR21).

## Conflict of Interest

The authors declare that the research was conducted in the absence of any commercial or financial relationships that could be construed as a potential conflict of interest.
