# Supplementary material for: Effects of Exercise on Depression, Anxiety, Cognitive Control, Craving, Physical Fitness and Quality of Life in Methamphetamine-Dependent Patients
Source: Front Psychiatry. 2020 Jan 28;10:999. doi: 10.3389/fpsyt.2019.00999 (PMC6997340; doi:10.3389/fpsyt.2019.00999)
Supplement: Supplementary file 1 [file Table_1.docx]

Table S1. Clinical evidence of physical exercise on methamphetamine addiction management

| Reference | Sample | Type of Exercise | Duration of exercise | Outcomes |
| --- | --- | --- | --- | --- |
| Dolezal et al., 2013  (1) | 29 MA-dependent individuals finished the proposed program: exercise training (ET, n = 15) or health education without training (equal attention [EA], n = 14). | Endurance Training(30 min): first 3 wks jogging and/or walking on treadmill during 30 min, at intensity based on heart rate (HR); the subsequent 5 wks had increasing intensity; Resistance Training(30 min): progressive, circuit-type resistance training program that included all the major muscle groups of the upper and lower body. | 3 d/wk, 8 wks | Aerobic capacity and endurance: VO_2_ max (↑21%);  Body composition and anthropometry: body fat (↓15%) and fat weight (↓18%) in the exercise group. |
| Dolezal et al., 2014  (2) | 50 recently abstinent MA-dependent participants with age-matched, drug-free controls (DF=22) and MA-dependent participants (MD = 28).  MD were randomized to thrice-weekly exercise training (ME = 14) or equal attention without training (MC = 14). | Endurance Training (30 min): first 3 wks jogging and/or walking on treadmill during 30 min, at intensity based on heart rate (HR); the subsequent 5 wks had increasing intensity; Resistance Training (30 min): progressive, circuit-type resistance training program that included all the major muscle groups of the upper and lower body. | 3 d/wk, 8 wks | Exercise markedly increased HRV: the ME group significantly increased SDNN (+34%), RMSSD (+63%), pNN50 (+173%), HFnu (+60%), and decreased HR (-7%), LFnu (-16%), and LF/HF (-19%);  Exercise improved in aerobic capacity (VO_2_ max; +24%);  Exercise increased muscle strength and endurance for upper; Body composition and anthropometry: body mass (−3%), percent relative body fat (−14%), and body mass index (−4%) in the exercise group. |
| Haglund et al., 2015  (3) | 135 MA-dependent individuals enrolled in residential treatment were randomly assigned to either a structured exercise intervention (n = 69) or a structured health education control group (n = 66). | Exercise sessions: 5 min of warm-up, 30 min of aerobic activity on a treadmill, 15 min of resistance training with weight-lifting in major muscle groups, and 5 min of cool-down and stretching. | 3 d/wk, 8 wks | Aerobic exercise intervention significantly reduced depression symptom scores. |
| Rawson et al. 2015a  (4) | 135 MA-dependent individuals were randomly assigned to exercise intervention (n = 69) or health education control (n = 66). | Exercise sessions: 5 min of warm-up, 30 min of aerobic activity on a treadmill, 15 min of weight training for the major muscle groups (arms, chest, back, and legs) and a 5 min cool-down with stretching. | 3 d/wk, 8 wks | Aerobic exercise significantly reduced depression and anxiety symptom scores (according to Beck Depression Inventory). |
| Rawson et al., 2015b  (5) | 135 individuals newly enrolled in treatment were randomly assigned to a structured 8-week exercise intervention (n = 69) or health education control group (n = 66). | Exercise sessions: 5 min of warm-up, 30 min of aerobic activity on a treadmill, 15 min of weight training for the major muscle groups (arms, chest, back, and legs) and a 5 min cool-down with stretching. | 3 d/wk, 8 wks | Physical exercise decreased MA use among lower severity MA users at 1-, 3-, and 6-months post treatment. This benefit was sustained for 6 months. |
| Robertson et al, 2016  (6) | 19 MA-dependent individuals were randomized to a supervised exercise training group (exercised; n = 10) or an equal-time health education training group (sedentary; n = 9). | Aerobic training (30 min): first 3 wks jogging and/or walking on treadmill during 30 min, at intensity based on HR; the subsequent 5 wks had increasing intensity;  Resistance training (30 min): progressive, circuit-type, resistance training that included all the major muscle groups of the upper and lower body. | 3 d/wk; 8 wks | Exercised patients displayed a significant increase in striatal D2/D3 receptor availability compared to the sedentary group; There were no changes in D2/D3 receptor availability in extra striatal regions in either group. |
| [Wang et al., 2015](#_ENREF_57)  (7) | 24 participants who met the DSM-IV criteria for MA dependence were randomly assigned to an exercise group or to a control group. | Aerobic exercise: 5 min warm-up, 20 min main exercise, 5 min cool-down.; During the main exercise, participants exercised on a stationary cycle ergometer (Monark 828E) at 50 RPMs; loading was manually adjusted to maintain the heart rate (HR) within 65% to 75% of the participant’s estimated maximum HR (i.e., 206.9-0.67 ×age). | acute aerobic exercise | The self-reported MA craving was significantly attenuated during, immediately following, and 50 min after the exercise session compared with the pre-exercise ratings, whereas the craving scores at these time points following exercise were lower than those for the reading control session; Acute exercise also facilitated inhibitory performance in both the standard and MA-related Go/NoGo tasks. |
| Wang et al., 2016  (8) | 92 individuals with MA dependence were randomly assigned to an exercise group (light, moderate, or vigorous intensity) or to a reading control group. | Aerobic exercise: 5-min warm-up; 20-min exercise using a bicycle ergometer at 50 rpm; 5-min cool down; Participants were instructed to cycle while keeping their HR at one of three desired exercise intensities: within the range of 40%-50%, 65%-75%, or 85%-95% of their maximum HR. | acute aerobic exercise | An inverted-U-shaped relationship between exercise intensity and inhibitory control was generally observed for the behavioral and neuroelectric indices, with the moderate intensity group exhibiting shorter Go reaction times, increased NoGo accuracy, and larger NoGo-N2 amplitudes. |
| [Wang et al., 2017](#_ENREF_59)  (9) | 62 people with MA dependencies recruited through the Drug Rehabilitation Bureau were assigned to either an aerobic exercise or attentional control group, with 50 participants completing the trial. | Moderate-intensity aerobic exercise (i.e., cycling, jogging, or jump rope) [65-75% of the indirect maximum heart rate (HRmax)]; Each session of the program involved stretching the large muscle groups as a warm-up (5 min), performing the aerobic exercise as the main exercise stage (30 min), and stretching the center-based muscle as a cool-down stage (5 min). | 3 d/wk, 12 wks | MA craving levels after 6 weeks of the exercise program, and the decreased trend was maintained until the termination of treatment; In the post-test, the exercise group also demonstrated more accuracy in behavioral inhibitory control as well as greater N2 amplitude in the NoGo condition of both the standard and MA-related tasks than those in the control group or pre-test. |
| Zhu et al., 2016  (10) | 60 male participants were allocated to the Tai Chi (n = 30) or standard care groups (n = 30) by administrative staff. | Tai Chi intervention: 10 min of warm-up, 30 min of Tai Chi practice, and 10 min of cool-down exercises. | 5 d/wk, 12 wks | Quality of life outcomes: Tai Chi group significantly increased in physiology, symptoms, society, and total score; Physical results showed a significant interaction with balance;  One-leg Stand with eye closed: the Tai Chi group improved by 10 s; The within-group factor displayed significant changes in body fat in both groups. |
| [Zhang et al., 2018](#_ENREF_63)  (11) | MA-dependent patients (n = 68) and healthy controls (n = 35); The MA-dependent patients were randomly divided into two groups: exercise group and non-exercise group. | Each session of the program involved stretching the large muscle groups as a warm-up (5 min), performing the aerobic exercise during the main phase (30 min), and stretching the center-based muscle during a cool-down stage (5 min). | 3 d/wk, 12 wks | Aerobic exercise improved the processing speed in MA-dependent patients;  aerobic exercise significantly attenuated a spontaneous increase in serum MDA levels in MA-dependent patients after 12-weeks of abstinence. |
